# Sero-prevalence of Hepatitis B virus surface antigen and associated factors among women of reproductive age in Bench Maji Zone, Southwest Ethiopia: community based cross-sectional study

**DOI:** 10.4314/ahs.v22i2.13

**Published:** 2022-06

**Authors:** Alemayehu Sayih Belay, Sisay Shewasinad Yehualashet, Dejene Derseh Abateneh, Kindie Mitiku Kebede

**Affiliations:** MizanTepi University, College of Health Sciences, Department of Nursing, MizanTeferi, P. O. Box 260, Bench Maji Zone, SNNPR, Ethiopia

**Keywords:** Ethiopia, Hepatitis B virus, Hepatitis B surface antigen, women, Reproductive age

## Abstract

**Background:**

Hepatitis B virus infection is one of the leading causes of liver diseases which occurs worldwide particularly in developing countries. It is often caused by prenatal transmission from mother to child or household transmission from a close contact during early childhood. It causes different complications like; jaundice, induces premature labor, and prematurity.

**Objective:**

The aim of this study was to estimate the sero-prevalence of hepatitis B virus surface antigen and associated factors among women of reproductive age in Bench Maji Zone, South West Ethiopia.

**Methods:**

A community-based cross-sectional study was conducted from December 15th, 2016 to February 15th, 2017. Multistage sampling technique was applied to select study participants. Logistic regression analysis was applied and p-values < 0.05 was used to see the significant association between dependent and independent variables.

**Results:**

A total of 330 participants were included in this study yielding 98.8% response rate. The sero-prevalence of HBsAg among women of reproductive age was 28(8.5%). Having multiple sexual partners (AOR = 18.73, 95% CI =3.65, 96.21) history of unprotected sex (AOR = 9.39, 95% CI =1.64, 53.77) were found to be significantly associated with sero-prevalence of HBV.

**Conclusions:**

The sero-prevalence of HBV infection among women of reproductive age was highly endemic. Hence, behavioral education and communication programs focusing on reduction of risky sexual behaviors should be designed to reduce HBV infection.

## Introduction

Hepatitis B virus (HBV) is a major public health problem which occurs worldwide particularly in developing countries. Hepatitis B virus is an infection which causes both acute and chronic hepatitis^1^–^3^. According to World Organization (WHO) 2013 report, 240 million people were thought to be chronically infected with hepatitis B virus; with the endemicity ranging from high (≥8%) to intermediate (2–7%) and low (<2%)^4^.

HBV is the leading cause of liver diseases like hepatocellular carcinoma (HCC) and cirrhosis worldwide^5^. The worldwide sero-prevalence of hepatitis B virus surface antigen (HBsAg) was estimated to be 3.61% in 2015^6^. The highest sero-prevalence of HBV (8 – 20%) was observed in countries of the African region^7^. It is estimated that 44% of cirrhotic disease and 47% of hepatocellular carcinoma (HCC) cases in sub-Sahara Africa are also attributed to HBV infection^8^.

In Africa and Asia, hepatitis B is often caused by prenatal transmission from mother to child or household transmission from close contact during early childhood. Acute hepatitis in pregnancy has been shown to cause different complications like; jaundice, induce premature labor, and prematurity.. The HBV status of the mother must be known before parturition or even before conception in order to prevent HBV transmission from mother to child^9^.

In Ethiopia, earlier hospital-based study showed that hepatitis B accounts for 12% of hospital admissions and 31% of deaths^10^. Another study in Ethiopia revealed that at least one of the hepatitis markers was found in 78% of patients with hepatocellular carcinoma, 86% of chronic hepatitis cases, and 88% of cirrhosis patients^11^.

Several studies on the sero-prevalence of Hepatitis B virus surface antigen among pregnant women who came for antenatal care were done in Ethiopia^12^–^14^. However, community based studies on the Sero-prevalence of Hepatitis B virus surface antigen among pregnant women in Ethiopia is rear. Therefore, the sero- prevalence and associated factors of HBV among pregnant women at the community level is hardly ever known in Ethiopia. Knowing the exact prevalence of HBV infection at the community level in developing countries like Ethiopia where there is low antenatal care (ANC) and institutional delivery, will be very helpful to decrease both maternal and fetal complications attributed to HBV infection^15^, ^16^. The use of community-based data may provide valuable insights about the burden of HBV infection at the community level in this study area where surveillance for HBV is limited. Therefore, this study was aimed at estimating the prevalence of hepatitis B virus surface antigen and associated factors among women of reproductive age at community level in Bench Maji Zone, South West Ethiopia.

## Methods

### Study design and setting

A community-based cross-sectional study design was done from December 15th, 2016 to February 15th, 2017. Bench-Maji is one of the Zones of the Southern Nations, Nationalities, and Peoples' Region (SNNPR) which is found 565 km away from the capital city Addis Ababa. Based on the 2007 census, the seven largest ethnic groups reported in this Zone were the Bench (45.11%), the Me'enit (21.36%), the Amhara (8.23%), the Kafficho (6.55%), the Dizi (5.17%), the Sheko (4.21%), and the Suri (3.88%). All other ethnic groups made up 5.49% of the population. This zone has a total of 11 districts; six of the districts (Debub Bench, Guraferda, Semen Bench, Shewa Bench, Sheko and Mizan-Aman town administration) are agrarian whereas, five of the districts (Bero, Maji, Meinit-Goldia, Meinit-Shasha and Surma) are and semi-pastoral districts.

### Sample size determination and sampling procedures

A single population proportion formula was used to estimate the sample size. The following assumptions were made while calculating the sample size; the margin of error d2= 0.03, Za/2= 1.96 and prevalence of HBV=0.03717. The final sample size was 334 after considering design effect of 2 and 10% non-response rate.

**Figure 1:**
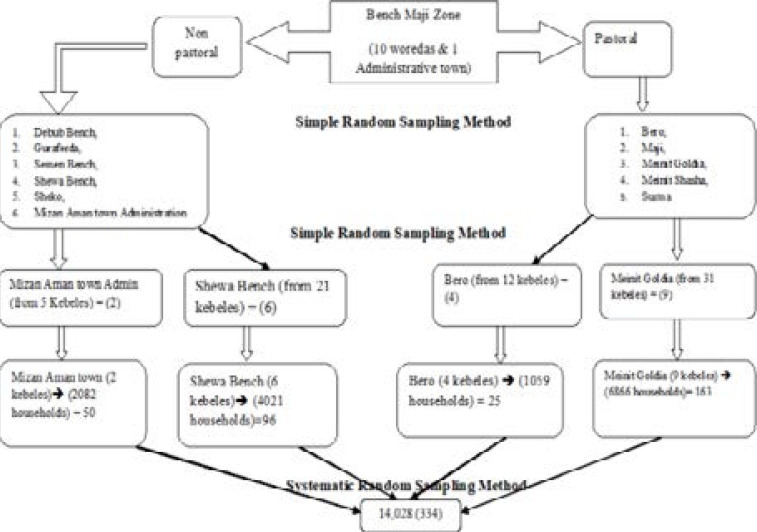
Schematic presentation of sampling procedure in Bench Maji Zone, South West Ethiopia, 2016/17

Multistage sampling technique was applied to select the study participants. After stratifying into agrarian and semi-pastoral districts, the primary sampling units (districts) were selected using simple random sampling method. Whereas, the secondary sampling unit (kebeles) and the tertiary sampling units (households) were selected using lottery method and systematic sampling method respectively. (Figure 1)

### Data collection tools and procedure

We used a structured interviewer administered questionnaire adapted from different peer-reviewed literatures17-19. The interview was carried out by 8 diploma holder laboratory technicians. The questionnaire was pretested among 5% of the total sample size in a similar set up before the actual data collection period and the necessary modifications and corrections were made to standardize and ensure the reliability of the questionnaire.

### Specimen collection and laboratory procedure

Upon completion of the questionnaire, capillary blood was collected through a skin puncture of the middle/ring finger by the sterile blood lancet. The collected whole blood sample was tested for sero-status of HBsAg using a commercial test strip (DiaSpot One Step Hepatitis B test Whole blood/serum/plasma, USA). The kit's performance ability is; sensitivity 100%, specificity 99.43%, and predictive value of a positive test 98.57%. The test was done according to the manufacturer's instructions and interpreted accordingly.

### Data quality assurance

The quality of data was assured by pre-tested and properly designed questionnaire. Furthermore, the data collectors were trained on data collection procedures.

The quality of the laboratory results was guaranteed by applying all quality control measures during sample collection and actual processing by following strict laboratory procedures. Standard operational procedures recommended by the manufacturers of advanced quality TM one step HBsAg test were strictly followed.

### Data processing and analysis

The data was entered using Epi Data Manager and was exported to statistical packages for social science (SPSS) version 21.0 for data cleaning and analysis.

Logistic regression analysis was applied and p-values ≤0.05 was used to see a significant association between dependent and independent variables. The results were presented in the form of tables, figures, and summary statistics.

### Ethical approval

Ethical clearance was obtained from the research directorate office of Mizan-Tepi University. After explaining the objectives of the study, written consent was obtained from each study participant. Interviews with study participants were conducted with strict privacy and assuring confidentiality. For those study participants whose results were positive, all the necessary information was provided, and finally, they were linked to the nearby health center.

## Results

### Socio-demographic Characteristics of the Respondents

From the total of 334 participants, 330 of them were interviewed which yields 98.8% response rate. The mean age of the participants was 32.56SD ±9.149. Nearly half of the participants, 135 (40.9%) were in the age range of 35–44 years.

The majority of respondents, 269 (81.5%) were rural residents. More than half of the participants, 209 (63.3%) were married. More than three fourth of respondents, 292 (88.5%) were housewives. (Table 1)

**Table 1:** Socio-demographic characteristics of the participants (n=330) in Bench Maji Zone, South West Ethiopia, 2016/17

Variables	Frequency	%
Place of residence		
Urban	61	18.5
Rural	269	81.5
Age of participants		
<=24	75	22.7
25–34	99	30.0
35–44	135	40.9
>=45	21	6.4
Marital status		
Married	209	63.3
Single	82	24.8
Divorced	19	5.8
Others*	20	6.1
Religion		
Protestant	146	44.2
Orthodox	130	39.4
Muslim	54	16.4
Ethnicity		
Bench	89	27.0
Meini't	56	17.0
Amhara	64	19.4
Oromo	35	10.6
Kaffa	36	10.9
Tigre	24	7.3
Others**	26	7.9
Respondent's educational status		
Unable to write and read	161	48.8
Able to read and write	69	20.9
Primary	83	25.2
Others***	17	5.2
Respondent's occupational status		
Housewife	292	88.5
Others****	38	11.5

* Separated and Widowed

** Dizzi and Sheka

*** Secondary, Certificate and above

**** Farmer, Merchant, Student, Daily worker, Government employee

### Characteristics of participants with its current HB-sAg sero-status

Among the total HBsAg positive participants, more than half 20 (71.4%) of them were unable to read and write, whereas about 5(17.9%) of sero-positive participants had family history of HBV infection. All 28(100) of sero-positive participants had no information about HBV infection and about 15(53.6) of them were ever lived with someone who has been diagnosed with hepatitis (Table 2)

**Table 2:** Participant's HBsAg status and related factors in Bench Maji Zone, Southwest Ethiopia, 2016/17

Variables	Current HBsAg Status	

	Positive N (%)	Negative N (%)
Educational status		
Unable to write and read	20(71.4)	141(46.7)
Able to read and write	6(21.4)	63(20.9)
Primary	2(7.1)	81(26.8)
Secondary	0(0)	6(2.0)
College and above	0(0)	11(3.6)
Family history of Hepatitis virus infection		
Yes	5(17.9)	0(0)
No	23(82.1)	302(100)
Ever lived with someone who has been diagnosed with		
hepatitis		
Yes	15(53.6)	1(0.3)
No	13(46.4)	301(99.7)
Information about HBV infection		
Yes	0(0)	15(5)
No	28(100)	287(95)

### Prevalence and Factors associated with hepatitis B virus infection

The overall prevalence of HBsAg was 8.5%95% CI (5, 12). Using bivariate logistic regression model; parity, gravidity, body piercing, contact with jaundiced person, having multiple sexual partner, unprotected sex, history of circumcision, and history of blood transfusion were significantly associated with current HBsAg status.

After adjusting possible confounders, having multiple sexual partners and unprotected sex were found to be independent predictors of HBV infection. Participants who had multiple sexual partner were almost 19 times more likely to be positive for HBV infection than those who didn't (AOR = 18.73, 95% CI =3.65, 96.21). Participants who had history of unprotected sex were almost 9 times more likely to be positive for HBV infection than those who didn't practice (AOR = 9.39, 95% CI =1.64, 53.77) (Table 3).

**Table 3:** Factors associated with HBV infection in Bench Maji Zone, Southwest Ethiopia, 2016/17

Variables	Current HBsAg Status		COR (95% C.I)	AOR (95% C.I)
	Negative N (%)	Positive N (%)		
Parity				
<=3	154(96.3)	6 (3.8)	**.014(.004–.051)** *	.100(.001–6.805)
3–5	143(94.7)	8(5.3)	**.020(.006–.069)** *	.473(.025–8.930)
> 5	5(26.3)	14(73.7)	1.00+	1.00+
Gravidity				
Primigravida	152(96.2)	6(3.8)	**.269 (.096–.756)** *	.441(.005–36.53)
Multi-gravida	75(87.2)	11(12.8)	1.00(.409–2.447)	.797(.097–6.542)
Grandmulti-gravida	75(87.2)	11(12.8)	1.00+	1.00+
Body piercing				
Yes	8(50.0)	8(50.0)	**14.70(4.99–43.27)** *	1.158(.099–13.568)
No	294(93.6)	20(6.4)	1.00+	1.00+
Contact with jaundiced person				
Yes	3(16.7)	15(83.3)	1.00+	1.00+
No	299(95.8)	13(4.2)	**.009(.002–.034)** *	.140(.006–3.105)
Multiple sexual partner				
Yes	71(75.5)	23(24.5)	**14.97(5.49–40.81)** *	**18.73(3.65–96.21)** *
No	231(97.9)	5(2.1)	1.00+	1.00+
Unprotected sex				
Yes	82(78.1)	23(21.9)	**12.34(4.54–33.54)** *	**9.39(1.64–53.77)** *
No	220(97.8)	5(2.2)	1.00+	1.00+
History of circumcision				
Yes	12(63.2)	7(36)	**8.06(2.87–22.6)** *	.867(.06–12.61)
No	290(93.2)	21(6.8)	1.00+	1.00+
History of blood transfusion				
Yes	1(33.3)	2(66.7)	1.00+	1.00+
No	301(92.0)	26(8.0)	**.043(.004–.492)** *	.039(.001–2.168)

* Adjusted for all significant variables p <0.05

+ Reference Category

## Discussions

In the current study, the overall prevalence of HBsAg was 8.5% with 95% C.I (5, 12). This finding is in consistent with other study findings in different parts of the world. For instance, the prevalence of HBsAg was found to be 10.8% in Yemen^20^, 9.3% in Kenya^21^, 5.49% in China, 22, and 6.9% in Nigeria^23^. However, the prevalence of HBsAg in this study area is higher than the prevalence of HBsAg observed in in Jimma (3.7%)^17^, in Dawuro (3.5%)^24^ and in Bahirdar (3.8%)^25^. The difference might be attributable to variations in socio-demographic cultural and behavioral related factors which are responsible for the risk of HBV infection.

In this study, participants with multiple sexual partners were almost 19 times more likely to be positive for HBV infection than participants who didn't have multiple sexual partners. This finding was found to be in line with studies conducted in Northwest Russia^26^, Sweden^27^ and Brazil^28^. Our finding was also similar with several studies conducted in different parts of Ethiopia like Dessie referral hospital^29^, Dawuro zone^24^, Deder hospital^30^, Arba Minch hospital^31^, Felege Hiwot referral hospital^32^ and Gandhi memorial hospital^33^. Sexual transmission has long been recognized as a major source of HBV transmission in the world^34^.

In addition to multiple sexual partners, unprotected sex was found to be an independent predictor of HBsAg. Participants who had a history of unprotected sex were almost 9 times more likely to be positive for HBV infection than who had not a history of unprotected sex. Studies from New York City^35^ and Rhode Island Hospital^36^ reported a similar findings. This similarity might be due to the fact that unsafe sex remains an important contributor to HBV transmission within early, advanced and regressing epidemics worldwide particularly, in Sub-Saharan Africa, and its social and behavioral factors play an important role in the transmission of HBV infection^37^–^39^. Moreover, in developing countries, different behavioral factors like; substance use, watching pornography^40^, and alcohol consumption^41^, were attributed to unprotected sex so that sex-related factors remain unquestionable reasons for the high prevalence of HBV infection.

This study has its own strength. First, the study was representatives of all women of reproductive age in the Bench-Maji zone covering all urban and rural residents. Second, laboratory-based data were done under strict quality control methods following standard operational procedure. However, this study has its own limitations. First, only surface antigen was identified since polymerase chain reaction test was not carried out. Second, there may befear to disclose their risky sexual behaviors. However, proper clarification about privacy and confidentiality was provided to each participants. Finally, we only included women of reproductive age group in the study. Therefore, the result needs to be interpreted with its' limitations.

## Conclusions

According to the WHO criteria, the current study showed that the burden of hepatitis B virus infection among reproductive age women was highly endemic. Behavioral risk factors such as having multiple sexual partners and history of unprotected sex may account for the high prevalence of hepatitis B virus infection among reproductive age women in this study area. Thus, in addition to immunization, screening, and treatment of HBV at health institutions, behavioral educations need to address these modifiable risk factors to reduce hepatitis B virus infection in this study area.

## List of abbreviations

HBsAg: Hepatitis B Virus Surface Antigen
HBV: Hepatitis B virus
HCC: Hepatocellular Carcinoma
MTU: MizanTepi University
PCR: Polymerase Chain Reaction
WHO: World Health Organization

## Declarations

### Availability of data and material

The datasets used and/or analyzed during the current study are available from the corresponding author on reasonable request.
